# Developing evidence-informed indicators to monitor HIV pre-exposure prophylaxis programmes across EU/EEA countries: a multi-stakeholder consensus

**DOI:** 10.2807/1560-7917.ES.2023.28.23.2200546

**Published:** 2023-06-08

**Authors:** Jef Vanhamel, Eline Wijstma, Jessika Deblonde, Marie Laga, Bea Vuylsteke, Christiana Nöstlinger, Teymur Noori

**Affiliations:** 1 Department of Public Health, Institute of Tropical Medicine, Antwerp, Belgium; 2 Department of Public Health and Surveillance, Sciensano, Brussels, Belgium; 3 Sexually Transmitted Infections, Blood-borne Viruses and Tuberculosis Section, European Centre for Disease Prevention and Control (ECDC), Stockholm, Sweden

**Keywords:** Human immunodeficiency virus, pre-exposure prophylaxis, PrEP, programmes, monitoring

## Abstract

Several countries in the European Union (EU) and European Economic Area (EEA) established and/or scaled up HIV pre-exposure prophylaxis (PrEP) programmes between 2016 and 2023. Data on PrEP programmes’ performance and effectiveness in reaching those most in need will be needed to assess regional progress in the roll-out of PrEP. However, there is a lack of commonly defined indicators for routine monitoring to allow for minimum comparability. We propose a harmonised PrEP monitoring approach for the EU/EEA, based on a systematic and evidence-informed consensus-building process involving a broad and multidisciplinary expert panel. We present a set of indicators, structured along relevant steps of an adapted PrEP care continuum, and offer a prioritisation based on the degree of consensus among the expert panel. We distinguish between ‘core’ indicators deemed essential for any PrEP programme in the EU/EEA, vs ‘supplementary’ and ‘optional’ indicators that provide meaningful data, yet where experts evaluated their feasibility for data collection and reporting as very context-dependent. By combining a standardised approach with strategic opportunities for adaptation and complementary research, this monitoring framework will contribute to assess the impact of PrEP on the HIV epidemic in Europe.

## The roll-out of HIV pre-exposure prophylaxis programmes in the EU/EEA

**Figure 1:**
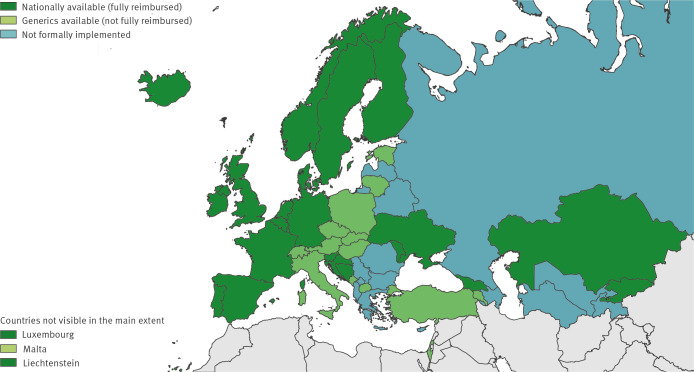
Status of formal PrEP implementation, World Health Organization European Region, 2022

Source: European Centre for Disease Prevention and Control Dublin Declaration Monitoring 2022 progress report [6].

In order to achieve the 95–95–95 Joint United Nations Programme on HIV and AIDS (UNAIDS) targets to end AIDS in the European Union (EU) and European Economic Area (EEA), scaling up combination HIV prevention programmes based on scientific evidence will be essential [1]. As efficacy and safety are high for the prophylactic use of oral antiretroviral medicines by people at substantial risk of HIV (i.e. PrEP), both the World Health Organization (WHO) and the European Centre for Disease Prevention and Control (ECDC) released a recommendation to include PrEP in countries’ existing HIV combination prevention packages [2-4]. After market authorisation for PrEP in the EU was granted in 2016, PrEP roll-out in the EU/EEA evolved rapidly [5]. France was the first country to officially provide PrEP in 2016. Data collected by the ECDC on implementation of the Dublin Declaration in the WHO European Region show that, from 2022, PrEP was reportedly available and fully reimbursed (i.e. through health insurance or public funds) in 18 of 30 EU/EEA countries (Figure 1) [1,6].

To translate the clinical efficacy of PrEP into population-level effectiveness, carefully designed and comprehensive programmes for PrEP are needed [7]. An effective PrEP programme is one in which people at substantial risk of HIV are adequately identified and offered PrEP through services that are acceptable to affected communities and in which PrEP users receive continued support for its correct use [8]. To achieve this, programmes need information on the epidemiological profile of potential PrEP candidates, while relying on available and appropriate infrastructure, staff and resources for PrEP care provision. Due to contextual differences in these elements, there is substantial variation between EU/EEA countries in the way PrEP is delivered and data are being collected, processed and reported [9].

In March 2021, the ECDC released an operational guidance with the goal to harmonise the approach to PrEP implementation [10]. This guidance outlined key principles for successful PrEP implementation, including quality statements and minimum standards. However, well-defined and uniform indicators to measure PrEP programmes’ performance using routine surveillance data are still lacking. We systematically developed a set of uniform indicators to monitor the roll-out of PrEP across EU/EEA countries, to allow for consistent reporting, enabling some comparability within the EU/EEA.

## The consensus-building process

Under coordination of the ECDC, a multidisciplinary research team with expertise in public health, monitoring and evaluation, and social sciences, was assembled to lead the process of developing a monitoring tool for PrEP. Its purpose was threefold: it should (i) support EU/EEA countries in identifying meaningful indicators for PrEP programme monitoring, (ii) offer insight into the anticipated benefits and challenges of using specific data sources to report on these indicators and (iii) recommend a minimum set of core indicators to be collected and reported in a harmonised way across the EU/EEA. To support the development of this tool, an expert panel was established. The ECDC coordination office identified and recruited experts within an existing network that supported the development of ECDC’s PrEP implementation guidance [10]. This network consisted of public health, clinical, academic and community experts from 22 EU/EEA countries. The research team together with the ECDC coordination office selected a purposive sample of panellists to ensure sufficient geographical representation as well as a diverse spectrum of expertise and perspectives relevant to the aim of the project. Experts were therefore selected based on their professional background and country of representation. Areas of expertise included: HIV/PrEP national routine surveillance systems, clinical practice in PrEP care, substantial HIV/PrEP experience through research, and civil society representation. The panel ultimately included 42 experts from 22 EU/EEA countries, Switzerland and Ukraine as well as representatives from UNAIDS, WHO/Europe, the International Union against Sexually Transmitted Infections (IUSTI), the European AIDS Clinical Society (EACS), Coalition Plus, IZKORAK and the European AIDS Treatment Group (EATG). See Supplement part 1 for additional details on the composition of the research team and expert panel.

The development of the tool consisted of two steps: a scoping review and country survey of available evidence, followed by a modified Delphi exercise to build consensus. 

**Figure 2:**
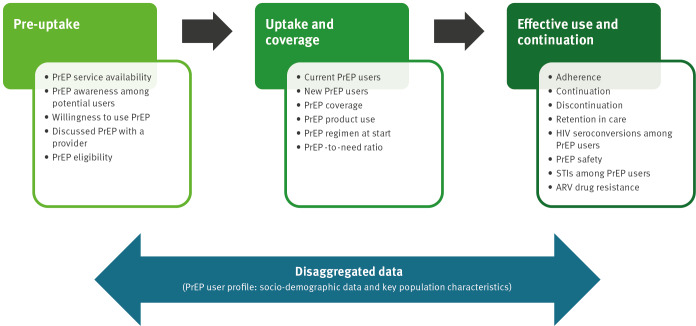
Evidence-informed candidate indicators organised along three key steps of the PrEP care continuum

For the scoping review, we conducted a systematic search of peer-reviewed and grey literature, to identify indicators currently used or suggested for monitoring PrEP programmes [11,12]. We complemented this systematic search with a rapid online survey, sent to one representative of the panel in each of 16 EU/EEA countries; we provide a copy of the country survey in Supplement part 2. In addition, these representatives could forward the survey to other self-identified PrEP experts in their country for review and to add complementary information. Each country representative could send back only one completed survey questionnaire to the research team. This questionnaire collected relevant practice-based evidence on the topic (e.g. existing PrEP monitoring activities, data sources and data availability) and explored the needs and expectations of different stakeholders of PrEP monitoring. We received 10 completed country questionnaires, based on contributions from 26 individual experts. Using a data matrix in MS Excel to combine the thematic analysis of qualitative data with the descriptive analysis of numerical data, we collated and synthesised the evidence from the scoping review and country survey to compile a list of candidate indicators for monitoring PrEP programmes. Inspired by a ‘prevention cascade’ approach, we organised the candidate indicators along three key steps of an adapted PrEP care continuum: (i) pre-uptake, (ii) uptake and coverage and (iii) effective use and continuation of PrEP (Figure 2) [13]. In addition, we included potential PrEP user characteristics for data disaggregation to reveal PrEP-related disparities. A full list of all candidate indicators with their accompanying definitions and a summary of useful data sources can be found in Supplement part 3.

PrEP: pre-exposure prophylaxis; STI: sexually transmitted infection; ARV: antiretroviral.

As a second step, we adopted a modified two-round Delphi technique to seek consensus among the expert panel on the importance and feasibility of implementing the identified candidate indicators in the EU/EEA context [14]. For more details on the methodological approach, we refer to Supplement part 4. Briefly, in a first online Delphi survey round, we asked expert panellists to quantitatively rate the perceived importance and feasibility of each candidate indicator and offered the possibility to add qualitative comments (e.g. contextualising feasibility issues with implementation and arguments for their importance). During a subsequent online meeting, we offered expert panellists the opportunity to discuss and adjust their ratings in group. This led to clarifying and refining indicator definitions to make them more accurate and context-adapted. In addition, experts discussed the possible added value and anticipated challenges for data collection and reporting. After this discussion, a second online Delphi survey was held among the meeting participants. Here, panellists voted to either include or exclude the discussed indicators in the final dataset, or they could provide final suggestions for indicator modification; see Supplement part 5 for a summary of the ratings for both Delphi survey rounds. After this final Delphi survey round, we integrated all feedback and resolved pending issues among a steering group consisting of the research team and five expert panellists. These panellists were selected based on relevant existing expertise with designing programmatic PrEP monitoring indicators, the status of PrEP implementation and monitoring in their country and representation from different professional backgrounds. For the list of names see Supplement part 1.

## The final set of indicators

The above systematic revision process led to adaptation and further refinement of the list of candidate indicators and ultimately enabled us to differentiate between three broader groups of indicators, each reflecting different levels of priority for reporting by EU/EEA countries. We refer to the Table for a full list of the indicators, their definitions and assigned level of priority.

**Table:** Final list of suggested PrEP monitoring indicators with their accompanying definitions and assigned priority label

Indicator name	Definition
Core	
Current PrEP users	The number of people who received PrEP at least once during the reporting period^a^
New PrEP users	The number of people who received PrEP for the first time in their lives during the reporting period^a^
Recent PrEP use among people newly diagnosed with HIV	The number of people who received PrEP at least once in the 12 months before being diagnosed with HIV among the people newly diagnosed with HIV during the reporting period^a^
Supplementary	
PrEP coverage	The number of people who used PrEP at least once during the reporting period^a^ among the estimated number of people who are eligible for PrEP according to local PrEP-eligibility criteria
PrEP continuation	The number of people who had at least one PrEP refill or follow-up visit in the 12 months after PrEP initiation among the number of people who were prescribed PrEP for the first time in their lives during the previous reporting period^a^
PrEP service availability	The number of facilities that offer PrEP per 100,000 population in a given geographical area within the country
Optional	
PrEP awareness among potential users	The number of people who report being aware of the existence of PrEP as an HIV prevention option among the number of people from a sample population who are questioned about PrEP awareness
Willingness to use PrEP	The number of individuals who report being willing to use PrEP if it were offered/available to them among the number of people from a sample population who are questioned about willingness to use PrEP

^a^ The reporting period for these indicators was set at 12 months (calendar year).

**Figure 3:**
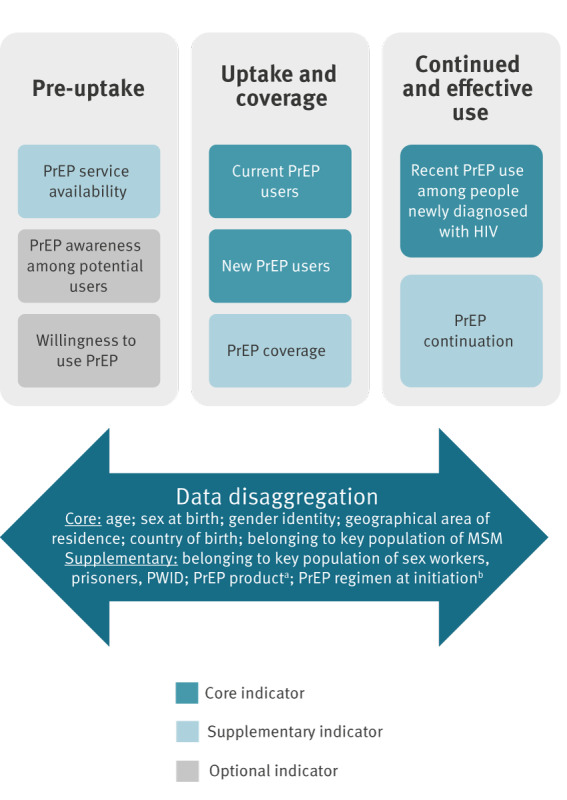
Final PrEP monitoring framework, organised along three key steps of the PrEP care continuum with priority label

First, the panellists achieved a strong consensus to include three indicators in a minimum core set. They found that these indicators conveyed essential information on key aspects of a PrEP programme’s performance and that any PrEP programme in the EU/EEA would be able to reported them. If tracked repeatedly, the number of *current PrEP users* may give an indication of the expansion of the programme over time, whereas the number of *new PrEP users* shows the programme’s ability to newly engage people into using PrEP (e.g. following demand creation activities). Importantly, as these two indicators may signal possible gaps in access to PrEP among certain population groups or in a given geographical area, the panel suggested to stratify them by relevant user characteristics. A distinction was made between core characteristics deemed essential for all countries in the EU/EEA, and supplementary characteristics to be considered depending on local context (Figure 3). As a proxy measure of effective use of PrEP, the panel promoted tracking recent PrEP use among people newly diagnosed with HIV. This indicator aims to direct attention to situations in which HIV seroconversions occurred despite demonstrated previous access to PrEP, flagging potential missed opportunities for HIV prevention. Experts acknowledged that outcomes of this indicator needed to be supplemented with further evaluation research to distinguish breakthrough infections under optimal adherence from possible programmatic issues, such as barriers to access, suboptimal adherence or discontinuation despite ongoing or fluctuating HIV risk.

PrEP: pre-exposure prophylaxis; MSM: men who have sex with men; PWID: people who inject drugs.

^a^ PrEP formulation refers to the type of PrEP product used (e.g. oral tenofovir disoproxil fumarate/emtricitabine vs injectable cabotegravir).

^b^ PrEP regimen refers to either a daily or non-daily (also called ‘event-driven’ or ‘on-demand’) regimen. The on-demand regimen can only be taken by cisgender men and transgender and gender-diverse people assigned male at birth who are not taking exogenous estradiol-based hormones.

In addition to the core set, the panel retained a set of supplementary indicators. They found *PrEP coverage* an important indicator to track a programme’s success in reaching the population who could benefit from PrEP. However, reliably estimating the size of the population in need of PrEP was perceived as complicated because criteria for eligibility differed across settings and there were barriers to routinely measuring behaviour associated with increased HIV risk. Although not a true measure of coverage, the *PrEP-to-need ratio* (PNR) was suggested as a pragmatic alternative to track the number of PrEP users relative to the number of new HIV diagnoses among a certain population group in a given area [15]. The panel agreed that the PNR is straightforward to construct and may reveal meaningful trends over time. Moreover, they deemed measuring *PrEP continuation* as relevant to evaluate whether clients are retained in follow-up as long as they use PrEP. Yet, the experts found it challenging to translate this concept into a single meaningful indicator for routine monitoring since prevention-effective PrEP use is not necessarily defined by uninterrupted longitudinal use. In addition, population-level databases may provide information on the volume of PrEP distributed at a certain time point, but not on how PrEP was actually dosed over time. Therefore, given that HIV risk is unlikely to change in the short term for a large number of people, focusing on whether at least one PrEP refill or follow-up visit was documented within 1 year after initiation might provide an indication of PrEP programmes’ success in supporting clients to engage with PrEP appropriately. The experts encouraged countries to complement the reporting on this indicator with additional implementation research into users’ reasons of discontinuing PrEP. Lastly, *PrEP service availability* could provide an indication of geographical proximity to facilities that offer PrEP, which is a dimension of access that may be particularly relevant for settings that organise PrEP follow-up through regular in-person visits.

We labelled a final group of indicators as ‘optional’, given that they all relied on the need to conduct repeated large surveys. Resources to conduct such studies may vary considerably between countries. Both indicators *PrEP awareness among potential users* and *willingness to use PrEP* shed light on the interest in PrEP in the community and may guide the planning of specific demand creation activities.

## Developing a pragmatic monitoring tool for PrEP programmes in the EU/EEA

We integrated the final list of retained indicators in a PrEP monitoring tool, providing PrEP programme implementers with a practical monitoring framework based on the PrEP care continuum (Figure 3); see [16] for a full report on the monitoring tool. We therefore developed indicator sheets that schematically provide an overview of the indicators’ priority level and that give a standardised definition, including numerator and denominator, where relevant. In addition, the indicator sheets present different options in terms of data sources to leave sufficient flexibility to collect relevant information to construct the indicators, briefly discussing their main strengths and limitations. Possible data sources include routine surveillance data (preferred), population size estimates and the use of specially designed or pre-existing surveys. The tool also suggests possible ways of triangulating multiple data sources to increase validity. Overall, this resulted in a pragmatic monitoring approach that supports countries in evidence-informed decisions for monitoring key steps of the PrEP care continuum adapted to their data collection and reporting capacity. 

## Addressing limitations of the monitoring tool

While a consensus approach for monitoring among EU/EEA countries increases consistency and comparability, it inevitably comes with trade-offs regarding its comprehensiveness. We acknowledge that this tool does not provide an exhaustive or normative list of what countries ought to monitor, yet it primarily aims to offer practical support for countries to identify and construct indicators in a meaningful way as they implement and scale up their PrEP programmes. To increase the local relevance of the tool, it highlights and encourages opportunities for synergies with further implementation research to obtain more accurate, granular and context-adapted insights. For example, reporting on *PrEP service availability* may provide an indication of geographical access to PrEP services when assessed per relevant administrative sub-unit (e.g. county, province or municipality), yet it does not take into account the capacity of individual facilities to meet the demand for PrEP. For this, the tool suggests themes and topics for additional evaluation research. A similar approach is proposed for the indicator on *PrEP continuation*, where deepened understanding into the patterns of PrEP use over time, through longitudinal research efforts, may reveal whether PrEP users are effectively and safely adapting PrEP use to periods of fluctuating HIV risk. We acknowledge that the tool in its current version is limited in that it does not propose indicators outside the scope of routine surveillance, such as on topics related to mental well-being or ‘quality of life’ of PrEP users. Nor does the tool disaggregate indicators by other relevant characteristics that were perceived by the panel as more challenging to streamline across countries, such as racial and/or ethnic background. The panel recommended that PrEP indicators should instead be disaggregated by migrant status, if data are available. Currently most countries collect information only on nationality or country of birth, while definitions used to characterise migrant and ethnic minority populations vary largely across countries. The panel emphasised, however, that data collection systems should be sensitive towards key populations’ intersecting vulnerabilities. Lastly, while this monitoring framework focuses on PrEP specifically, it should be viewed as part of a broader combination prevention approach that may involve other options such as condom use. In the monitoring tool, opportunities for integration with existing monitoring systems are therefore highlighted.

## Conclusion

This evidence-informed and expert-led programmatic PrEP monitoring tool is an important first step towards harmonising the EU/EEA’s approach to monitoring key steps in the roll-out of PrEP and exploring its impact on ending AIDS in Europe. This tool is meant to be a pragmatic guidance that countries can use flexibly in different phases of PrEP roll-out. Users of the tool are thus encouraged to actively engage with the provided suggestions and make adaptations where necessary. A context-sensitive routine monitoring approach that combines multiple data sources, complemented with additional insights from research and evaluation, will be needed to reflect the complex reality wherein PrEP users may start, stop, re-start and – in the future – switch PrEP products based on individual convenience and preference.

## Supplementary Data

530000 Supplement
