# Supplementary material for: Developing evidence-informed indicators to monitor HIV pre-exposure prophylaxis programmes across EU/EEA countries: a multi-stakeholder consensus
Source: Euro Surveill. 2023 Jun 8;28(23):2200546. doi: 10.2807/1560-7917.ES.2023.28.23.2200546 (PMC10318945; doi:10.2807/1560-7917.ES.2023.28.23.2200546)
Supplement: Supplement [file 22-00546_VANHAMEL_Supplement.pdf]

Supplementary materials

This supplementary material is hosted by Eurosurveillance as supporting information alongside the article "Developing evidence-informed indicators to monitor HIV pre-exposure prophylaxis programmes across EU/EEA countries: a multi-stakeholder consensus", on behalf of the authors, who remain responsible for the accuracy and appropriateness of the content. The same standards for ethics, copyright, attributions and permissions as for the article apply. Supplements are not edited by Eurosurveillance and the journal is not responsible for the maintenance of any links or email addresses provided therein.

Supplementary material 1: Composition of research team and expert panel.

1. Research team

NAME	COUNTRY/ORGANISATION
1. Jef Vanhamel	Belgium (ITM Antwerp)
2. Eline Wijstma	Belgium (ITM Antwerp)
3. Jessika Deblonde	Belgium (Sciensano Brussels)
4. Marie Laga	Belgium (ITM Antwerp)
5. Bea Vuylsteke	Belgium (ITM Antwerp)
6. Christiana Nöstlinger	Belgium (ITM Antwerp)

2. Expert panel

NAME	COUNTRY/ORGANISATION
1. Jessika Deblonde	Belgium
2. Josip Begovac	Croatia
3. Anna Kubátová	Czech Republic
4. Henrikki Brummer-Korvenkontio	Finland
5. Jean-Michel Molina	France
6. Jérémy Zeggagh	France
7. Uwe Koppe	Germany
8. Binod Mahanty	Germany

9. Daniel Schmidt	Germany
10. Ioannis Mameletzis	Greece
11. Caroline Hurley	Ireland
12. Fiona Lyons	Ireland
13. Carole Devaux	Luxembourg
14. Valeska Padovese	Malta
15. Silke David	Netherlands
16. Elske Hoornenborg	Netherlands
17. Birgit Van Benthem	Netherlands/STI CC
18. Alma Cicic	Montenegro
19. Milena Stevanovikj	North Macedonia
20. Arild Johan Myrberg	Norway
21. Justyna Kowalska	Poland
22. Milosz Parczewski	Poland
23. Margarida Tavares	Portugal
24. Claudia Estcourt	Scotland
25. Janez Tomažič	Slovenia
26. Julia Del Amo	Spain
27. Asuncion Diaz	Spain
28. Pep Coll	Spain
29. Finn Filen	Sweden
30. Benjamin Hampel	Switzerland
31. Natalie Messerli	Switzerland
32. Matthias Reinacher	Switzerland
33. Olga Denisiuk	Ukraine
34. Ann Sullivan	EACS
35. Antons Mozalevskis	WHO Regional Office for Europe
36. Rosalind Coleman	UNAIDS
37. Raj Patel	IUSTI
38. Andrew Winter	IUSTI
39. Jürgen Rockstroh	EACS
40. Daniela Rojas Castro	Coalition Plus
41. Gus Cairns	EATG
42. Zoran Dominković	IZORAK

3. Steering group

NAME	COUNTRY/ORGANISATION
1. Jef Vanhamel	Belgium (ITM Antwerp)
2. Eline Wijstma	Belgium (ITM Antwerp)
3. Jessika Deblonde	Belgium (Sciensano Brussels)
4. Marie Laga	Belgium (ITM Antwerp)
5. Bea Vuylsteke	Belgium (ITM Antwerp)
6. Christiana Nöstlinger	Belgium (ITM Antwerp)
7. Teymur Noori	ECDC
8. Claudia Estcourt	IUSTI
9. Ann Sullivan	EACS
10. Zoran Dominković	IZKORAK
11. Uwe Koppe	Germany (RKI)
12. Silke David	The Netherlands (RIVM)

Supplementary material 2: Rapid online country survey.

	1. UPDATE COUNTRY CASE STUDY*
	This section aims to capture the latest updates regarding the status of PrEP implementation in your country. You will find the country case study that you submitted previously here, to assist you to fill in this section. If more options are possible, please tick all that apply.
A	**What is the current status of PrEP implementation in your country?**
	☐National program with co-payments<br>☐National program without co-payments<br>☐Private prescription and online purchase<br>☐Ongoing pilot or research project<br>☐Other: please specify
B	**Are there any major changes in the main service delivery approach for PrEP** [i.e. type of provider and delivery setting of PrEP]**, compared to the country case study? If yes, please specify. If no, please proceed to section 2.**

2. DATA ON PrEP INITIATIONS

In this section we will focus on available data on ***people who initiate PrEP*** *(i.e. first-time PrEP users or 'PrEP starters') in your country.*

<table>
<tr><td rowspan="2">A</td><td colspan="2">How many people have started PrEP (i.e. have received PrEP for the first time in their lives) in the year 2019[1] in your country? Please specify how this number was produced.

If no estimate on national level can be provided, please provide any other (more peripheral) available data on PrEP starters and specify the source(s). If no data at all are available, please proceed to question C.</td></tr>
<tr><td colspan="2"></td></tr>
<tr><td rowspan="5">B</td><td colspan="2">Please specify for each level (i.e. national/regional, and facility-level), <u>all possible available data sources</u> that you are currently aware of and that could inform the number of PrEP starters in your country. If any (ongoing) research is available that could inform this number, please specify.

- Possible sources can include (but are not limited to): (sub-)national surveillance, clinical record data (i.e. patient data), pharmacy data (e.g. boxes of TDF/FTC sold), claims data (e.g. reimbursed prescriptions/consultations for PrEP), etc.
- (Sub-)national or regional level concerns the availability of data covering the entire country (or county/region for sub-national level), either through central data collection systems, or through the reporting of peripherally collected data (i.e. at facility-level) to a (sub-)national focal point.
- Facility-level concerns the availability of data at the specific facility or site where they are collected and/or registered (i.e. at pharmacy or at certain clinics).</td></tr>
<tr><td>Level (national/regional, facility) and research data</td><td>Data sources (e.g. surveillance, clinical records, pharmacy data, claims data, other?). Please specify.</td></tr>
<tr><td>1. National or regional (if applicable)</td><td></td></tr>
<tr><td>2. Facility level</td><td></td></tr>
<tr><td>3. Research data (or ongoing research projects) Please specify.</td><td></td></tr>
</table>

[1] We request data on the year 2019 as data on the reporting year of 2020 might not be available yet in some settings and/or COVID-19 might have caused considerable disruptions in data collection/registration compared to regular reporting years. However, if only data outside the requested time span are available, then please provide these data and specify the reporting period.

**C**	**What are some of the main challenges you currently encounter with regard to monitoring the number of PrEP initiations in your country?** [i.e. potential for over- versus under-reporting (accuracy), possibility that certain groups or regions are not included (coverage) etc.] **Please explain briefly.**

**D**	**Please indicate whether the following information on PrEP starters is – to your knowledge – available in your country? If yes, can you explain briefly (e.g. data sources, national/regional and facility-level etc.) and provide an estimate if possible/available?**				
	**Category**		**Available?**	**Can you specify? (e.g. available data sources, facility vs. national/regional level etc.)**	**If available, estimate for 2019[1].**
	Sex		☐YES<br>☐NO<br>☐ Don't know		
	Age (group)		☐YES<br>☐NO<br>☐ Don't know		
	Country of birth		☐YES<br>☐NO<br>☐ Don't know		
	**KEY POPULATION**	MSM	☐YES<br>☐NO<br>☐ Don't know		
		Transgender people	☐YES<br>☐NO<br>☐ Don't know		
		Sex workers	☐YES<br>☐NO<br>☐ Don't know		
		People who inject drugs	☐YES<br>☐NO<br>☐ Don't know		
		Prisoners	☐YES<br>☐NO<br>☐ Don't know		

	PrEP regimen (daily versus event-driven)	☐YES<br>☐NO<br>☐ Don't know		
	Setting where PrEP was prescribed (e.g. HIV clinic, private practice, general hospital etc.)	☐YES<br>☐NO<br>☐ Don't know		

3. ADDITIONAL DATA RELATED TO PrEP

In this section we will focus on additional PrEP data (other than 'PrEP starters') currently available in your country.

A — **To your knowledge, are any data on the following topics related to PrEP available in your country? If yes, specify the indicator used and its data source, and provide an estimate if possible/available.**

Category	Available?	Specify (e.g. indicator used + data source)	If available, estimate for 2019[1].
Number of people eligible for PrEP (e.g. per key population).	☐YES<br>☐NO<br>☐ Don't know		
Number of people having received PrEP at least once.	☐YES<br>☐NO<br>☐ Don't know		
Number of people that discontinued PrEP.	☐YES<br>☐NO<br>☐ Don't know		
Number of HIV seroconversions among people previously prescribed PrEP.	☐YES<br>☐NO<br>☐ Don't know		
Any other relevant information on PrEP use?	N/A		

4. FUTURE PERSPECTIVES FOR MONITORING PrEP

A — **To your knowledge, are there any (other) potential data sources that are currently unexploited/under-used in your country and that could support the monitoring of PrEP in the future?**

B	**According to you, what are key priorities for improving the monitoring of PrEP in your country? (please list at least three in descending order of importance, i.e. number 1 being the most important one)**
	1.<br>2.<br>3.
C	**What are your expectations/wishes from this project? What kind of output/tool would add the most value to you at this stage of monitoring PrEP rollout in your country?**

**5. FINAL COMMENTS**	
A	**Please share below any other information, comments and/or suggestions that could be relevant regarding the topic of monitoring PrEP (either for your country or at the EU/EEA level).**

* This survey was sent out to representatives of 16 different EU/EEA countries: Belgium, Croatia, Czech Republic, England, France, Germany, Ireland, Malta, The Netherlands, Norway, Poland, Scotland, Slovenia, Spain, Sweden and Switzerland. We received 11 completed questionnaires, from: Belgium, Croatia, England, France, Germany, Ireland, Malta, The Netherlands, Scotland and Spain.

Supplementary material 3: list of candidate indicators and accompanying definitions, and a list of useful data sources for PrEP monitoring.

Indicator name	Description
**1. PrEP service availability**	The number of PrEP providers in a given area or per 100.000 population.
**2. PrEP awareness**	The number of people who report being aware of the existence of PrEP as an HIV prevention option, regardless of whether or not it is available to them.
**3. Willingness to use PrEP**	The number of people who report being willing to use PrEP if it were offered/available to them.
**4. Discussed PrEP with provider**	The proportion of PrEP-eligible people that discussed PrEP with a provider, e.g. requested or received information on PrEP.
**5. PrEP eligibility**	The number of people that are (estimated to be) eligible for PrEP, according to local PrEP eligibility criteria.

**6. Number of sexual health service attendees**	The number of people that attended a sexual health service (SHS) in the reporting period (e.g. 12 months).
**7. Current PrEP use**	The number of people who used PrEP **at least once** during the reporting period (e.g. last 12 months).
**8. New PrEP initiations**	The number of people who initiated PrEP for the first time in their lives (i.e. who were previously naïve to the use of ARVs for the purpose of HIV prevention) during the reporting period (e.g. the last 12 months).
**9. Profile of current PrEP users**	The number of people who used PrEP **at least once** during the reporting period (e.g. last 12 months), disaggregated by some key characteristics of these users.
	Disaggregations: sex, age, key population (MSM, transgender persons, PWID, sex workers, prisoners), nationality, country of birth, socio-economic status, place of residence.
**10. PrEP coverage**	The number of people who used PrEP at least once during the reporting period (e.g. 12 months), divided by the size of the total PrEP-eligible population.
**11. PrEP product use**	The number of people who used PrEP at least once during the reporting period, stratified by specific PrEP product choice (i.e. oral TDF/FTC, oral TDF/3TC, oral TDF, or – in the future – injectable Cabotegravir or oral Islatavir).
**12. PrEP dosing regimen**	The proportion of people taking PrEP on a daily versus event-driven basis during the reporting period.
**13. PrEP-to-need ratio**	The number of people who used PrEP at least once during the reporting period in a given area (e.g. 12 months), divided by the number of new HIV diagnoses during the reporting period (e.g. 12 months).
**14. Adherence**	The proportion of PrEP users with sufficient or good adherence to the chosen PrEP dosing regimen (i.e. who take pills as directed to have protective drug levels in the blood during condomless intercourse).
**15. PrEP continuation**	The proportion of people who initiated PrEP and continued taking PrEP until a certain time point.
**16. PrEP discontinuation**	The number of people who are reported to have stopped PrEP OR who did not return for a follow-up visit during the reporting period (and who still received PrEP in the previous reporting period).
**17. Retention in care**	The number of HIV tests performed among all PrEP users during the reporting period (e.g. 12 months), divided by the number of HIV tests expected to have been performed among all PrEP users during the reporting period (e.g. 12 months).
**18. HIV seroconversions among PrEP users**	The number of new HIV diagnoses among people prescribed PrEP at least once during the reporting period and who had at least one follow-up HIV test.
**19. PrEP safety**	The number and type of (serious) adverse events that occur among PrEP users during the reporting period and are related to PrEP use.
**20. STI diagnoses**	The number of new diagnoses of chlamydia, gonorrhoea and syphilis among PrEP users during the reporting period.
**21. ARV resistance**	The proportion of blood specimens from PrEP users or people with a history of PrEP use and diagnosed with HIV that were tested and which show genetic mutations associated with (ineffective) PrEP use.

Table 1. List of useful data sources for monitoring programmatic PrEP indicators, as identified in this scoping review and rapid country survey.

Data source	PrEP indicators that can be constructed using this data source	Reported Benefits	Reported Challenges	Comments
**Pharmacy prescription database**	Number of PrEP users;<br>Number of PrEP initiations (if UICs available);<br>PrEP continuation (if UICs available);<br>PrEP adherence as the proportion of days covered by a prescription (if UICs available);	1) Algorithms applied to the specified databases can identify TDF/FTC prescriptions for PrEP with high sensitivity and high specificity (after excluding indications for HIV infection, HBV infection or TDF/FTC for PEP)<br><br>2) Readily available data<br><br>3) Population-level data	1) Databases not exhaustive (e.g., unavailability of data from prescriptions through closed health systems)<br><br>2) Possible lack of insight into the proportion of missing data or whether missingness is differential<br><br>3) Limited sociodemographic data (e.g., on sex, age and place of residence, but not KP)	Provides estimates on prescriptions *written*
**Pharmacy dispensation database**				Provides estimates on prescriptions *filled*
**Medical claims database**	Number of PrEP users;<br>Number of PrEP initiations (if UICs available);<br>PrEP continuation (if UICs available);<br>Retention in PrEP services (i.e. through HIV testing as proxy measure);<br>HIV infection (e.g., through HIV testing followed by ART prescription)<br>PrEP adherence as the proportion of days covered by a prescription (if UICs available);			Provides estimates on prescriptions *filled*
**PrEP facility/clinic registries**	PrEP need (i.e. proportion of clinic attendants who report behaviour consistent with PrEP-eligibility)<br>Number of PrEP initiations;<br>Number of PrEP users;<br>Sociodemographic data (including KP)<br>Dosing regimen;<br>PrEP adherence, as self-reported or as the proportion of days covered by a prescription;<br>Continuation;<br>Discontinuation and reasons;<br>Retention in PrEP services;<br>New STI diagnoses	1) Not approximations, but records.<br><br>2) Data can be kept at the client-level	1) Incomplete data if administrative burden on facility staff is high	Using standardized service codes facilitates aggregation of local data for national estimates

**National HIV surveillance**	New HIV diagnoses among total population;<br>New HIV diagnoses among PrEP users (if data can be linked to PrEP databases)<br>Proxy for PrEP need (i.e. denominator for PrEP-to-need ratio)	1) Population-level data	1) Limited sociodemographic characteristics<br><br>2) Unique identifier codes are needed to link HIV diagnoses to PrEP use.	/
**Repeated surveys**	PrEP awareness;<br>Willingness to use PrEP;<br>PrEP need (i.e. proportion of respondents who report behaviour consistent with PrEP eligibility);<br>Estimates of PrEP use prevalence (among respondents);<br>Trends in the profile of PrEP users (although surveys likely target only 1 KP);<br>Estimates of dosing regimen;<br>Estimates of adherence;<br>Estimates of continuation;<br>Estimates of discontinuation and reasons;<br>Changes in behaviour after PrEP initiation;<br>Trends in STI diagnoses;	1) Survey questions are adaptable to local contexts<br><br>2) Behaviour, knowledge and attitudes cannot be not captured by other data sources	1) Surveys likely comprise convenience samples, and results may consequently not be generalizable to the total population<br><br>2) Self-reported outcomes are susceptible to information bias, including recall bias and social desirability bias<br><br>3) Financial and human resources are required to disseminate, (administer), and analyze surveys	To date, large-scale behavioural surveys to monitor PrEP have focused on MSM.<br><br>Different sampling methods (e.g., venue-based or internet-based) may yield characteristically different population samples.<br><br>Generally, internet-based surveys are more timely and lower cost than in-person surveys and have a broader geographic scope.

Supplementary material 4: methodological details of the Delphi process.

1. The Delphi technique

In order to facilitate the process of ultimately finding the most reliable consensus on indicators suitable to include in a practical monitoring tool for PrEP programmes in the EU/EEA, we used a modified Delphi method as an overall guiding approach[2]. The Delphi technique has been characterized as "*a method for structuring a group communication process so that the process is effective in allowing a group of individuals, as a whole, to deal with a complex problem*"[3]. Key advantages of using the technique are (1) the ability to build consensus in areas where scientific evidence is insufficient and/or conflicting, (2) the possibility to involve a wide range of experts from many countries at distance, with their indirect interaction being mediated by the study team, and (3) the ability to 'level-out' the influence of relatively dominant voices when using regular discussion groups. The modified Delphi method applied in this project existed of two phases, and combined a comprehensive review of existing evidence on the topic of PrEP monitoring (phase 1) with the collective judgement of a multidisciplinary expert panel (phase 2).

To this extent, an international expert panel was established with support of ECDC, consisting of 42 members. This panel included people from different backgrounds (clinical, research, and community), yet all with practice-based experience with the implementation and/or monitoring of PrEP or related programmes, and representing different EU/EEA Member States (see supplementary material 1 for the full list of experts). This expert panel committed to participating to the entire consensus-building process.

2. Consensus-building process

The data collection phase consisted of two online survey rounds, with an online meeting of the expert panel in-between survey rounds. Data collection took place between September and November 2021. We used Formsite as online survey platform, and Webex as online videoconference tool. The first Delphi survey was conducted in September 2021 and consisted of the 21 candidate indicators identified through the evidence review phase. Expert panelists were asked to rate the perceived importance and feasibility of implementing the candidate indicators in their respective contexts on a 7-point Likert scale (1=not important or not feasible at all; 7=very important or very feasible). Experts were asked to base their ratings on the accompanying evidence summary tables (see Table 1 and Table 2), combined with their personal opinion and experience. Participating experts had the opportunity to provide additional qualitative comments for each indicator, to clarify their ratings or to express particular concerns they might have had. A link to the online survey can be found here.

In total, 30 out of 42 invited experts completed the first online survey. For each candidate indicator, we calculated the experts' median rating for importance and feasibility, and the percentage of experts rating on either extremes of the scale (i.e. 6-7 as top rating, and 1-3 as bottom rating).

Based on the ratings of the experts, the research team categorized the candidate indicators into their likelihood of being included in a future monitoring tool. The following algorithm was used to distinguish between indicators that could be (1) included without further discussion, (2) for which inclusion was uncertain, or (3) indicators that would be eligible for exclusion due to poor ratings of importance and feasibility:

---

[2] McCorry NK, O'Connor S, Leemans K, et al. Quality indicators for Palliative Day Services: A modified Delphi study. *Palliative Medicine*. 2019;33(2):197-205. doi:10.1177/0269216318810601.

[3] Chitu Okoli, Suzanne D. Pawlowski. The Delphi method as a research tool: an example, design considerations and applications. Information & Management. 2004; 42 (1) : 15-29. doi.org/10.1016/j.im.2003.11.002.

- INCLUDE WITH NO DISCUSSION: Median importance rating of 6-7 with NO DISAGREEMENT
  - Disagreement = 25% or more experts rated 1-3 on importance scale OR expressed substantial reservations in the qualitative comments
- UNCERTAIN: Median importance rating of 3-5 (regardless of agreement) OR median importance rating of 6-7 WITH DISAGREEMENT
  - Disagreement = 25% or more experts rated 1-3 on importance scale OR expressed substantial reservations in qualitative comments
- EXCLUDE: median importance rating of 1-2 (regardless of agreement) OR indicators from uncertain category that remain unresolved after discussion

The online meeting with the expert panel was held on 9 November 2021. The aim of the meeting was to:

1. Agree to include those indicators in the monitoring tool that have a high median score of importance (6-7) with no disagreement.
2. Agree to discard indicators deemed of insufficient importance (median importance score of 1-2).
3. Discuss the appropriateness of including those indicators in the monitoring tool that fall under the 'uncertainty' category.
4. Discuss which indicators could be part of a minimum common 'core set' of indicators to be collected and reported across the EU/EEA.

After plenary discussion, during which indicators were refined to make them more accurate and adapted to the EU context, attending panellists were asked to vote for final inclusion or exclusion of the (refined) indicator in the monitoring tool, distinguishing between inclusion in a common 'core set' to be reported across EU/EEA MSs and relevant indicators to adapt on a country-level basis. The cut-off to include indicators in the final tool was set as follows:

- INCLUDE: if 75% or more of panellists voted for either inclusion or inclusion of a modified indicator (and provided suggestions for modification).
- EXCLUDE: if >25% of panellists votes for exclusion of indicator.

See supplementary material 4 for a synthesis of expert panellists' ratings.

Pending issues were resolved in a steering group consisting of the research team, the ECDC coordinator and 5 expert panellists (see Supplementary Material 1). These panellists were selected based on their particular expertise with designing programmatic PrEP monitoring indicators (i.e. one expert led a previous publication on the topic), the status of PrEP implementation and monitoring in the country (including both representation from countries more advanced in PrEP monitoring as well as "starters"), and representation from different professional backgrounds (clinical, public health

surveillance, and civil society) in addition to those of the research team (epidemiological, social sciences and M&E).

Supplementary material 5: synthesis of the expert panel's ratings for both Delphi survey rounds.

Indicator name	Description	Number of panellists who rated the *importance* of monitoring the specified indicator as 1 to 7, during the first Delphi survey<br><br>1=not at all important; 7=extremely important<br>Median score is indicated by the red bar<br><br>(N of participating panellists = 30)	Percentage of panellists who voted to include or exclude the specified indicator in a monitoring tool, during the second Delphi survey (*after plenary discussion*)<br><br>(N of participating panellists = 31)	
			**As supplementary indicator**<br><br>(= implementation up to individual EU/EEA MSs)	**As core indicator**<br><br>(= to be collected systematically by all EU/EEA MSs)
**1. Current PrEP use**	The number of people who used PrEP **at least once** during the reporting period (e.g. last 12 months)	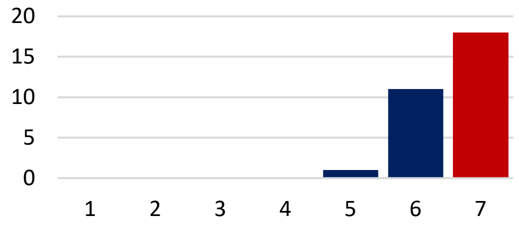	N/A	Include as defined: 82.6 %<br>Include modified indicator: 17.4 %<br>Exclude: 0.0 %<br>Outcome: retained as core indicator.
**2. New PrEP initiations**	The number of people who initiated PrEP for the first time in their lives (i.e., who were previously naïve to the use of ARVs for the purpose of HIV prevention) during the reporting period (e.g. the last 12 months).	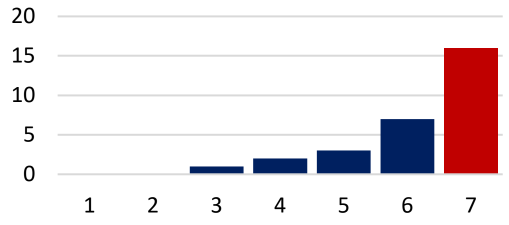	N/A	Include as defined: 87.5 %<br>Include modified indicator: 8.3 %<br>Exclude: 4.2 %<br>Outcome: retained as core indicator.

**3. PrEP product use**	The number of people who used PrEP at least once during the reporting period, stratified by specific PrEP product choice (i.e. oral TDF/FTC, oral TDF/3TC, oral TDF, or – in the future – injectable Cabotegravir or oral Islatavir).	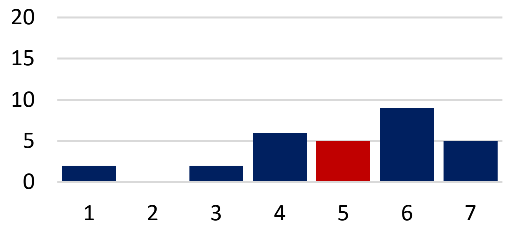	Include as defined: 52.0 %<br>Include modified* indicator: 20.0 %<br>Exclude: 28.0 %<br>*Only if relevant in the specific context (i.e., if multiple PrEP products are available)<br>Outcome: excluded as separate indicator; PrEP product added as relevant disaggregator for PrEP use (i.e. PrEP use by product).	N/A
**4. PrEP dosing regimen**	The proportion of people taking PrEP on a daily versus event-driven basis during the reporting period.	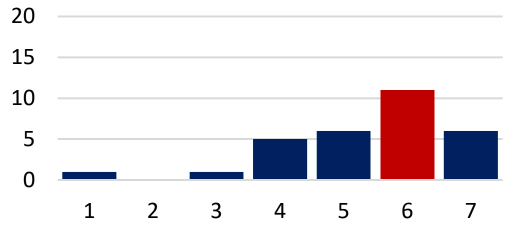	Include as defined: 37.0 %<br>Include modified* indicator: 33.3 %<br>Exclude: 29.6 %<br>*The proportion of people taking PrEP on a daily versus *non-daily* basis during the reporting period.<br>Outcome: excluded as separate indicator; dosing regimen added as relevant disaggregator for PrEP use at start (i.e. chosen PrEP dosing regimen at initiation).	N/A
**5. Number of sexual health service attendees**	The number of people that attended a sexual health service (SHS) in the reporting period (e.g. 12 months)	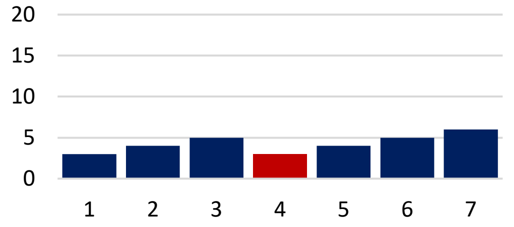	Outcome: Excluded as separate indicator; not discussed during Delphi session 2 – specified proxy for PrEP need perceived to be unsuitable in many (non-UK) contexts	

**6. PrEP coverage**	The number of people who used PrEP at least once during the reporting period (e.g. 12 months), divided by the size of the total PrEP-eligible population.	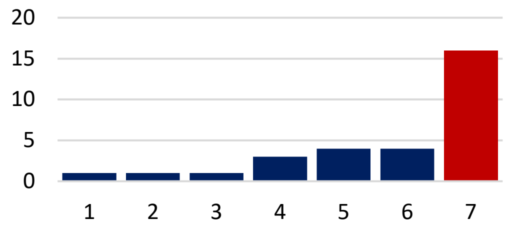	N/A	Include as defined: 22.7 %<br>Include modified* indicator: 9.1 %<br>Exclude: 68.2 %<br>*For the denominator: define 'PrEP-eligibility' according to local criteria; monitor PrEP coverage only if it is expected that valid estimates of the population in need of PrEP can be made<br>Outcome: retained as supplementary indicator given context-dependent challenges defining denominator (i.e. eligible population).
**7. PrEP-to-need ratio**	The number of people who used PrEP at least once during the reporting period in a given area (e.g. 12 months), divided by the number of new HIV diagnoses during the reporting period (e.g. 12 months).	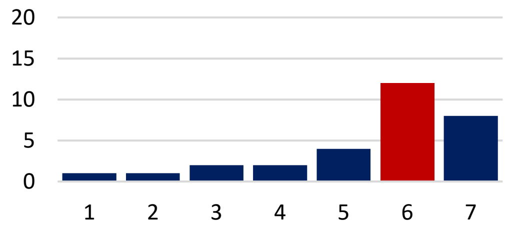	N/A	Include as defined: 45.5 %<br>Include modified* indicator: 45.5 %<br>Exclude: 9.1 %<br>*Advise to estimate the PrEP-to-need ratio across the EU/EEA in research first; If outcomes are meaningful and helpful, re-consider incorporating this indicator in routine monitoring.<br>Outcome: retained as alternative supplementary indicator for PrEP coverage

<table>
<tr>
<td>8. Adherence</td>
<td>The proportion of PrEP users with sufficient or good adherence to the chosen PrEP dosing regimen (i.e. who take pills as directed to have protective drug levels in the blood during condomless intercourse).</td>
<td>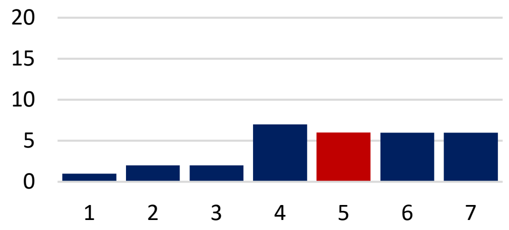
</td>
<td><table>
<tr><td>Include as defined</td><td>16.7 %</td></tr>
<tr><td>Include modified indicator</td><td>8.3 %</td></tr>
<tr><td>Exclude</td><td>75.0 %</td></tr>
</table>
Outcome: excluded as separate indicator as panel perceived it more as a clinical monitoring indicator (not programmatic).</td>
<td><table>
<tr><td>Include as defined</td><td>9.5 %</td></tr>
<tr><td>Include modified indicator</td><td>4.8 %</td></tr>
<tr><td>Exclude</td><td>85.7 %</td></tr>
</table></td>
</tr>
<tr>
<td>9. PrEP continuation</td>
<td>The proportion of people who initiated PrEP and continued taking PrEP until a certain time point.</td>
<td>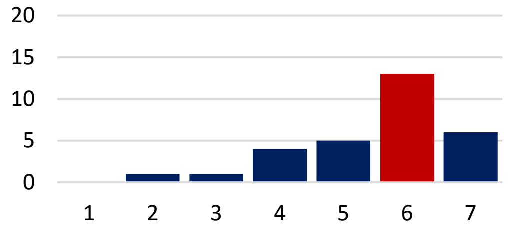
</td>
<td><table>
<tr><td>Include as defined</td><td>20.8 %</td></tr>
<tr><td>Include modified* indicator</td><td>16.7 %</td></tr>
<tr><td>Exclude</td><td>62.5 %</td></tr>
</table>
*Align continuation indicator with (local) PrEP follow-up guidelines
Outcome: retained as supplementary indicator.</td>
<td><table>
<tr><td>Include as defined</td><td>19.0 %</td></tr>
<tr><td>Include modified* indicator</td><td>14.3 %</td></tr>
<tr><td>Exclude</td><td>66.7 %</td></tr>
</table></td>
</tr>
<tr>
<td>10. PrEP discontinuation</td>
<td>The number of people who are reported to have stopped PrEP OR who did not return for a follow-up visit during the reporting period (and who still received PrEP in the previous reporting period).</td>
<td>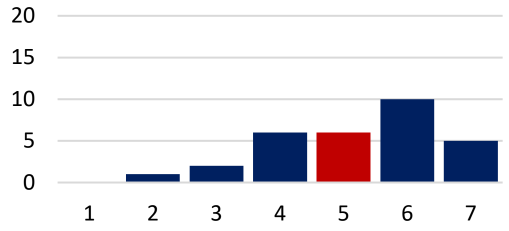
</td>
<td><table>
<tr><td>Include as defined</td><td>29.2 %</td></tr>
<tr><td>Include modified* indicator</td><td>20.8 %</td></tr>
<tr><td>Exclude</td><td>50.0 %</td></tr>
</table>
*Disaggregated by whether an individual is lost-to-follow-up, and/or the reason for PrEP discontinuation.
Outcome: excluded as separate indicator; merged with “PrEP continuation” indicator.</td>
<td><table>
<tr><td>Include as defined</td><td>40.9 %</td></tr>
<tr><td>Include modified indicator</td><td>0.0 %</td></tr>
<tr><td>Exclude</td><td>59.1 %</td></tr>
</table></td>
</tr>
</table>

<table>
<tr>
<td>11. Retention in care</td>
<td>The number of HIV tests performed among all PrEP users during the reporting period (e.g. 12 months), divided by the number of HIV tests expected to have been performed among all PrEP users during the reporting period (e.g. 12 months).</td>
<td>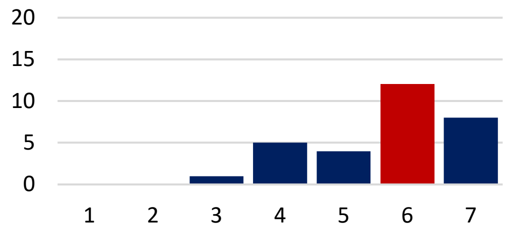
</td>
<td><table><tr><td>Include as defined</td><td>34.8 %</td></tr><tr><td>Include modified* indicator</td><td>26.1 %</td></tr><tr><td>Exclude</td><td>39.1 %</td></tr></table>
*Preference to base retention on follow-up visit attendance, rather than HIV testing adherence

Outcome: excluded as separate indicator due to lack of accuracy and relevance.</td>
<td><table><tr><td>Include as defined</td><td>35.0 %</td></tr><tr><td>Include modified* indicator</td><td>10.0 %</td></tr><tr><td>Exclude</td><td>55.0 %</td></tr></table>
*Preference to base retention on follow-up visit attendance, rather than HIV testing adherence</td>
</tr>
<tr>
<td>12. New HIV diagnoses among PrEP users</td>
<td>The number of new HIV diagnoses among people prescribed PrEP at least once during the reporting period and who had at least one follow-up HIV test.</td>
<td>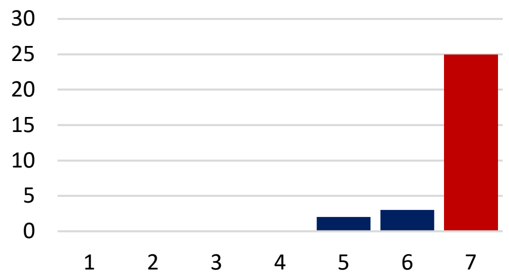
</td>
<td>N/A</td>
<td><table><tr><td>Include as defined</td><td>54.2 %</td></tr><tr><td>Include modified* indicator</td><td>41.7 %</td></tr><tr><td>Exclude</td><td>4.2 %</td></tr></table>
*New HIV diagnoses among people who ever used PrEP, disaggregated whether or not the individual recently used PrEP (i.e., in the last 12 months)

<u>Outcome: refined indicator retained as core indicator.</u></td>
</tr>
<tr>
<td>13. STI diagnoses</td>
<td>The number of new diagnoses of chlamydia, gonorrhoea and syphilis among PrEP users during the reporting period.</td>
<td>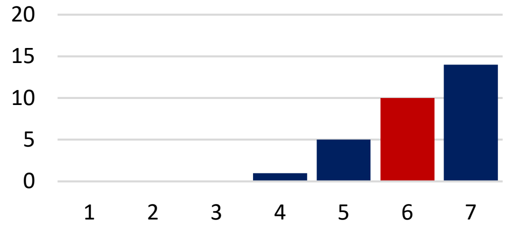
</td>
<td>N/A</td>
<td><table><tr><td>Include as defined</td><td>59.1 %</td></tr><tr><td>Include modified* indicator</td><td>22.7 %</td></tr><tr><td>Exclude</td><td>18.2 %</td></tr></table>
*Number of PrEP users newly diagnosed with chlamydia, gonorrhoea, syphilis or Hepatitis C during the reporting period.

Outcome: excluded as separate indicator due to outside scope of PrEP monitoring tool as such;</td>
</tr>
</table>

<table>
<tr><td></td><td></td><td></td><td></td><td>suggestion included to integrate PrEP monitoring with STI monitoring systems.</td></tr>
<tr><td>14. PrEP safety</td><td>The number and type of (serious) adverse events that occur among PrEP users during the reporting period and are related to PrEP use.</td><td>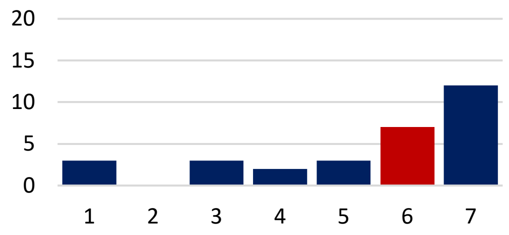
</td><td colspan="2">Outcome: excluded as separate indicator; not discussed during Delphi session 2 – to be linked to general pharmacovigilance monitoring</td></tr>
<tr><td>15. ARV resistance</td><td>The proportion of blood specimens from PrEP users or people with a history of PrEP use and diagnosed with HIV that were tested and which show genetic mutations associated with (ineffective) PrEP use.</td><td>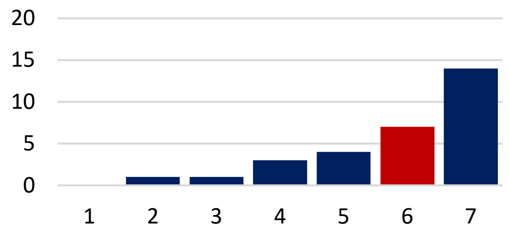
</td><td colspan="2">Outcome: excluded as separate indicator; not discussed during Delphi session 2 – to be linked to general HIV monitoring</td></tr>
<tr><td>16. PrEP service availability</td><td>The number of PrEP providers in a given area or per 100.000 population.</td><td>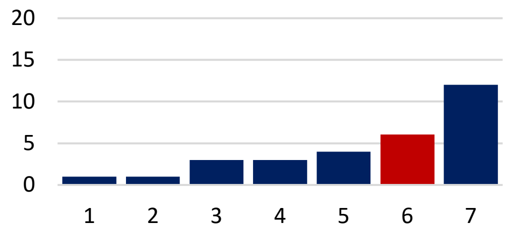
</td><td><table><tr><td>Include as defined</td><td>79.2 %</td></tr><tr><td>Include modified indicator</td><td>12.5 %</td></tr><tr><td>Exclude</td><td>8.3 %</td></tr></table>
Outcome: indicator retained as supplementary indicator.</td><td>N/A</td></tr>
</table>

**17. PrEP awareness**<br><br>**Via surveys - research**	The number of people who report being aware of the existence of PrEP as an HIV prevention option, regardless of whether or not it is available to them.	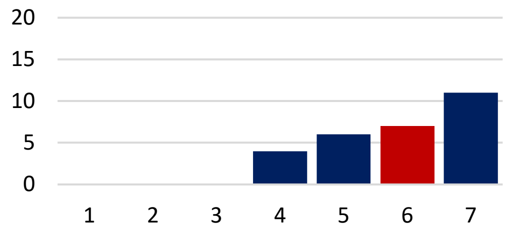	Include as defined: 22.7 %<br>Include modified indicator: 18.2 %<br>Exclude: 59.1 %<br><br>Outcome: indicator retained as optional indicator given outside routine surveillance (e.g. need for special surveys).	N/A
**18. Willingness to use PrEP**<br><br>Via surveys - research	The number of people who report being willing to use PrEP if it were offered/available to them.	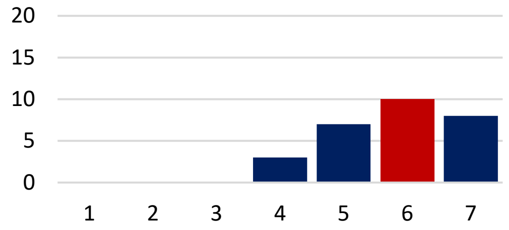	Include as defined: 18.2 %<br>Include modified indicator: 18.2 %<br>Exclude: 63.6 %<br><br>Outcome: indicator retained as optional indicator given outside routine surveillance (e.g. need for special surveys).	N/A
**19. Discussed PrEP with provider**<br><br>Via surveys – research (or facility data)	The proportion of PrEP-eligible people that discussed PrEP with a provider, e.g. requested or received information on PrEP.	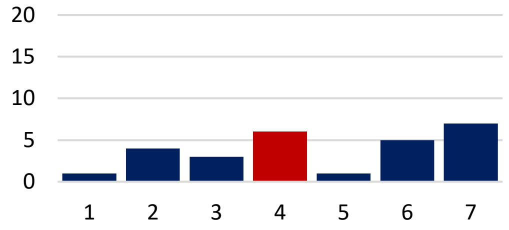	Include as defined: 9.5 %<br>Include modified indicator: 9.5 %<br>Exclude: 81.0 %<br><br>Outcome: excluded as separate indicator due to lack of relevance and feasibility.	N/A

**20. Eligibility**	The number of people that are (estimated to be) eligible for PrEP, according to local PrEP eligibility criteria.	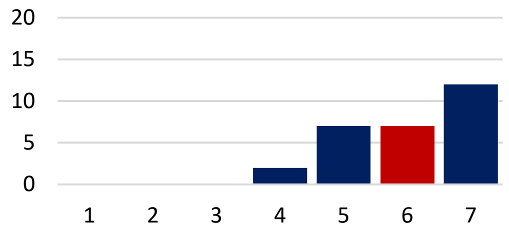	Include as defined: 15.8 %<br>Include modified* indicator: 52.6 %<br>Exclude: 31.6 %<br>*Devise a (nationally relevant) algorithm or proxy to predict the size of the PrEP-eligible population based on data of – among other possible predictors- STI incidence and HIV incidence.<br><br>Outcome: excluded as separate indicator; integrated in denominator of "PrEP coverage" indicator.	N/A
**21. Profile of current PrEP users**	The number of people who used PrEP **at least once** during the reporting period (e.g. last 12 months), disaggregated by some key characteristics of these users.<br><br>Disaggregations: sex, age, key population (MSM, transgender persons, PWID, sex workers, prisoners), nationality, country of birth, socio-economic status, place of residence (geographical location).	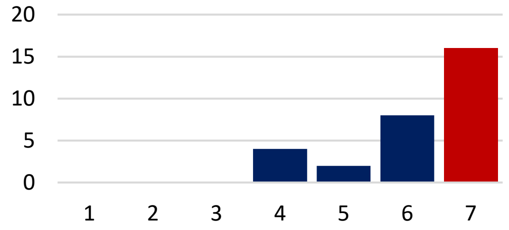	**Sex at birth**<br>N/A	Include as defined: 52.4 %<br>Include modified* indicator: 47.6 %<br>Exclude: 0.0 %<br>*Sex at birth AND gender identity (m/f/non-binary/other)<br><br>Outcome: retained as core disaggregator.
			**Age**<br>N/A	Include as defined: 95.2 %<br>Include modified indicator: 0.0 %<br>Exclude: 4.8 %<br>Outcome: retained as core disaggregator.

<table>
<tr><td rowspan="7">Profile of current PrEP users, continued…</td><td rowspan="7"></td><td rowspan="7"></td><td colspan="2">Belonging to the KP of MSM</td></tr>
<tr><td>N/A</td><td>Include as defined: 85.7 %
Include modified indicator: 9.5%
Exclude: 4.8 %
Outcome: retained as core disaggregator.</td></tr>
<tr><td colspan="2">Belonging to the KP of transgender people (TGP)</td></tr>
<tr><td>N/A</td><td>Include as defined: 38.1 %
Include modified* indicator: 52.4 %
Exclude: 9.5 %
*do not ‘collect’ separately, but base indicator on sex at birth and gender identity; disaggregate by transgender men and transgender women
Outcome: exclude as separate indicator; derive from combining disaggregators “gender” and “sex at birth”</td></tr>
<tr><td colspan="2">Belonging to the KP of people who inject drugs (PWID)</td></tr>
<tr><td>N/A</td><td>Include as defined: 66.7 %
Include modified* indicator: 23.8 %
Exclude: 9.5 %
*use of any drug (including chemsex)
Outcome: retain as supplementary disaggregator.</td></tr>
<tr><td colspan="2">Belonging to the KP of sex workers (SW)</td></tr>
</table>

Profile of current PrEP users, continued…			N/A	Include as defined 45.0 %<br>Include modified* indicator 30.0 %<br>Exclude 25.0 %<br>*report PrEP use among male MSW and FSW separately<br><br>Outcome: retain as supplementary disaggragator.
			Belonging to the KP of prisoners	
			Include as defined 42.3 %<br>Include modified* indicator 0.0 %<br>Exclude 57.7 %<br>Outcome: retained as supplementary disaggregator.	N/A
			Country of birth (as indicator for migration status)	
			Include as defined 96.3 %<br>Include modified* indicator 0.0 %<br>Exclude 3.7 %	Include as defined 95.2 %<br>Include modified* indicator 0.0 %<br>Exclude 4.8 %<br>Outcome: retained as core disaggregator.
			Nationality	
			Include as defined 22.2 %<br>Include modified* indicator 7.4 %<br>Exclude 70.4 %<br>Outcome: retained as supplementary disaggregator	N/A

			(in case country of birth not available).	
			Place of residence (geographical location)	
			Include as defined 63.0 %<br>Include modified* indicator 14.8 %<br>Exclude 22.2 %<br>Outcome: retained as supplementary disaggregator.	N/A
			Socioeconomic status (SES)	
			Include as defined 12.9 %<br>Include modified* indicator 67.7 %<br>Exclude 19.4 %<br>*Replace SES with 'highest completed education'<br>Outcome: excluded as separate indicator; no single best SES disaggregator, to be judged in context which SES indicator is most relevant to report.	N/A
